# Characteristics of sauna deaths in Korea in relation to different blood alcohol concentrations

**DOI:** 10.1007/s12024-018-9993-7

**Published:** 2018-06-21

**Authors:** Kyung-Moo Yang, Bong-Woo Lee, Jaeseong Oh, Seong Ho Yoo

**Affiliations:** 1 Division of Forensic Medicine, National Forensic Service, Seoul, South Korea; 2 Medical Examiner’s Office, National Forensic Service, Wonju, South Korea; 3 Department of Clinical Pharmacology and Therapeutics, Seoul National University College of Medicine and Hospital, Seoul, South Korea; 4 Department of Forensic Medicine and Institute of Forensic Medicine, Seoul National University College of Medicine, Seoul, South Korea

**Keywords:** Autopsy, Blood alcohol concentration, Intoxication, Sauna death

## Abstract

Although the benefits of sauna bathing have been demonstrated in epidemiological studies, sauna deaths have been reported. The aim of this study was to determine the demographic and forensic characteristics associated with different blood alcohol concentrations (BACs) in sauna deaths in Korea. In this retrospective analysis, data were collected from a nationwide pool in Korea between January 2008 and December 2015 to determine the role of alcohol intoxication in sauna deaths based on the subjects’ BAC and to evaluate the demographic and forensic characteristics associated with different BACs. One hundred and three deaths were classified into 2 groups: the non-intoxication (NI) group (BAC,<0.08%; *n* = 27) and the intoxication (I) group (BAC,≥0.08%; *n* = 76). Demographic and forensic characteristics were compared between the groups using a multinomial logistic regression analysis. The proportions of decedents who were male (odds ratio: 17.4, 95.0% confidence interval: 3.8–79.8) and in a prone position at the scene of death (odds ratio: 11.3, 95.0% confidence interval: 2.1–60.1) were significantly higher (*P* < 0.001 and *P* < 0.05, retrospectively) in the I group than in the NI group. However, no significant differences were observed with respect to obesity, coronary artery narrowing, and liver pathology. Sauna deaths exhibited different characteristics according to BACs detected at autopsy. The differences in sauna deaths between the I and NI groups may have implications for the targeted prevention of sauna deaths associated with alcohol consumption.

## Introduction

Sauna bathing is a cultural tradition in Finland. Regular sauna bathing is associated with a reduced risk of sudden cardiac death, fatal coronary heart disease, and all-cause mortality among the general population in Finland [1]. In cardiovascular disease cohorts in Japan [2], repeated sauna bathing has been shown to be associated with a reduction in systolic blood pressure without affecting heart rate in patients with chronic congestive heart failure.

Traditionally, Koreans have believed that a hot sauna relieves fatigue and cures a hangover. In Korea, there are many jjimjilbangs. Jjimjilis derived from the Korean word meaning “heating” and bang is derived from that meaning “room.”. Jjimjilbangs are public bathhouses furnished with showers, hot tubs, and a sauna, that have become part of the modern lifestyle in Korea. Jjimjilbangs provide hot sauna rooms with temperatures ranging from 80.0–110.0 °C (similar to Finland) to suit guests who prefer relaxing temperatures. The sauna practices at jjimjilbangs are different from those in Finland in that many Koreans rest lying down on the sauna floor (which is made of stone or wood), because of the traditional custom of lying down on an ondol (stone floor) without beds. Therefore, sauna victims are unresponsive in the event of an unwitnessed collapse. Due to the popularity of Korean jjimjilbangs, there is a risk of sauna deaths. Although most large series investigating sauna deaths have been conducted in Finland and Sweden [3–6], forensic pathologists in Korea have also investigated sauna deaths in jjimjilbangs. Finnish and Swedish data suggest that cardiovascular disease is the underlying cause of death in most natural deaths in saunas. A high prevalence of alcohol consumption was also reported in sauna deaths. However, considering the small number of reports on the beneficial effect of hot sauna bathing in for those with cardiovascular disease [1, 2], the hazardous role of alcohol consumption in sauna deaths has not be fully discussed. Moreover, in studies of sauna deaths [3–6], the authors have not stratified blood alcohol concentrations (BACs) into non-intoxication (NI) and intoxication (I) groups. Therefore, to investigate the characteristics of acute alcohol intoxication in sauna deaths, we divided the cases of sauna deaths into two groups based on BAC: the NI group (BAC, <0.08%) and the I group (BAC, ≥0.08%) according to BACs defined by the National Institute on Alcohol Abuse and Alcoholism.

The aim of this study was to investigate the demographic and forensic characteristics associated with different BACs in sauna deaths in Korea.

## Materials and methods

### Patients and samples

In this retrospective analysis, data were collected from a nationwide pool of sauna deaths at jjimjilbangs in Korea between January 2008 and December 2015.The inclusion criterion was individuals who had died in a sauna room. The exclusion criterion was individuals who had died in a room in the immediate vicinity of a sauna room (e.g. washroom, hot tub, or dressing room). One hundred and three (0.3%) of the 31,123 autopsy cases identified over the 8-year study period met the inclusion criterion. Data were collected from forensic autopsy reports, all of which included toxicological testing for drugs and alcohol, as well as, police reports of the individuals’ previous medical records. Toxicological specimens were obtained from heart blood from the right ventricle or peripheral blood from the femoral vein. Drug screening tests were performed using gas-chromatography and mass spectrometry. Blood alcohol and carboxyhemoglobin concentrations (to assess carbon monoxide intoxication) were analyzed by headspace gas chromatography. Information regarding body position at the scene of death (in a sauna) was obtained from the police reports. The body mass indexes (BMI) of the 103 individuals were stratified into 4 categories, according to the revised Asia-Pacific BMI criteria by the World Health Organization Western Pacific Region [7], as follows: underweight (BMI < 18.5 kg/m^2^), normal (BMI ≥ 18.5 and < 23.0 kg/m^2^), overweight (BMI ≥ 23.0 and < 25.0 kg/m^2^), and obese (BMI ≥ 25.0 kg/m^2^). The extent of coronary atherosclerosis was determined by reporting forensic pathologists as minimal, mild, moderate, or severe based on cross-sectional luminal narrowing (stenosis) by atherosclerotic plaques: minimal (luminal narrowing <25.0%), mild (luminal narrowing 25.0–50.0%), moderate (luminal narrowing 50.0–75.0%), and severe (luminal narrowing>75.0%), as described previously [8]. The overall reported extent of coronary artery disease was considered the most advanced level of atherosclerosis from all 3 major coronary artery branches (right coronary artery, left anterior descending artery, and left circumflex artery). Liver pathology was classified by microscopic examination as none, fatty liver disease, or liver cirrhosis, based on microscopic findings of pathological reports.

### Statistical analyses

All variables were analyzed using descriptive statistics. Bivariate analysis of comparisons between the NI and I groups was performed using Chi-square tests or Fisher’s exact tests. Multinomial logistic regression analysis was performed to evaluate differences in characteristics between the 2 groups. The NI group was used as the reference category to evaluate differentially associated factors in sauna deaths according to BAC. Independent variables included demographic (age, sex, and obesity) and forensic characteristics (coronary artery narrowing, liver pathology, and body position at the scene of death). All statistical analyses were conducted using Statistical Package for the Social Sciences for Windows (software version 23.0; SPSS Inc., Chicago, IL, USA). A *P* < 0.05 was considered statistically significant.

## Results

**Fig. 1:**
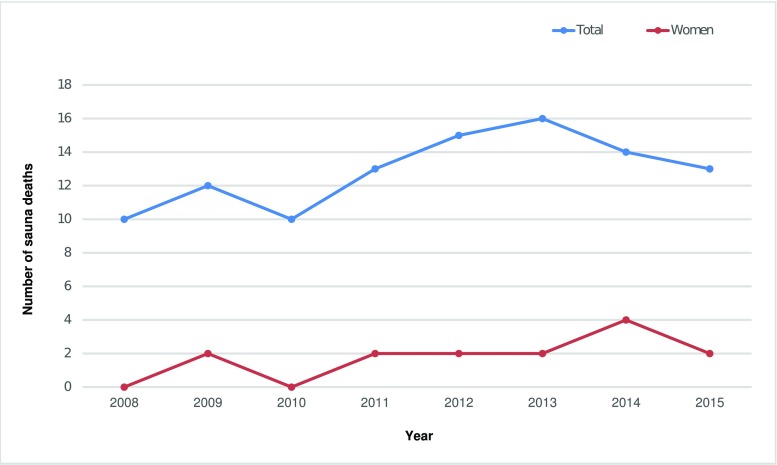
Annual distribution of autopsy-diagnosed sauna deaths

Between January 2008 and December 2015, 103 deaths were recorded in hot sauna rooms at jjimjilbangs in Korea. Figure 1 shows the annual distribution of deaths that occurred in hot saunas during the study period. A slightly increasing trend was observed after 2010.

Table 1 summarizes the demographic and forensic characteristics of the 103 sauna deaths. All the individuals were Asian-Korean. Eighty-eight (85.4%) were men and 15 (14.6%) were women. The mean age was 55 (range, 26–86) years. Thirty-three (32.0%) were aged 50–59 years and 33 (32.0%) were aged >60 years. Forty-six men (52.3%) and 12 women (80.0%) were aged >50 years. Among the decedents, 29 (28.2%) were obese, 30 (29.1%) overweight, 40 (38.8%) normal weight, and 4 (3.9%) underweight according to BMI. The extent of coronary atherosclerosis was minimal in 50 individuals (48.5%), mild in 23 individuals (22.3%), moderate in 11 individuals (10.7%), and severe in 19 individuals (18.5%). From microscopic examination of the liver, 87 individuals (84.5%) had no pathology, 15 (14.6%) had fatty liver disease, and 1 (0.9%) had liver cirrhosis.

**Table 1:** Demographic and forensic characteristics of autopsied sauna decedents

Characteristic	Decedents (*n* = 103)
Sex, *n* (%)	
Female	15 (14.6)
Male	88 (85.4)
Age (years), *n* (%)	
< 29	2 (1.9)
30–39	6 (5.9)
40–49	29 (28.2)
50–59	33 (32.0)
≥ 60	33 (32.0)
Obesity, *n* (%)	
Underweight(BMI <18.5 kg/m^2^)	4 (3.9)
Normal (BMI ≥18.5 and < 23.0 kg/m^2^)	40 (38.8)
Overweight (BMI ≥23.0 and < 25.0 kg/m^2^)	30 (29.1)
Obese (BMI ≥25 kg/m^2^)	29 (28.2)
Coronary artery narrowing, *n* (%)	
Minimal	50 (48.5)
Mild	23 (22.3)
Moderate	11 (10.7)
Severe	19 (18.5)
Liver pathology, *n* (%)	
None	87 (84.5)
Fatty liver disease	15 (14.6)
Liver cirrhosis	1 (0.9)
Position, *n* (%)	
Supine	50 (48.6)
Prone	37 (35.9)
Side	10 (9.7)
Sitting	6 (5.8)
BAC (%), *n* (%)	
< 0.01	22 (21.4)
0.01–0.08	5 (4.8)
≥ 0.08	76 (73.8)
Cause of death, *n*(%)	
Non-heart disease-related	27 (26.2)
Heart disease-related	76 (73.8)

*BAC* blood alcohol concentration, *BMI* body mass index

The manner of death was classified as due to accidental causes in 13 individuals (12.6%) (hyperthermia [*n* = 9] and acute ethanol intoxication [*n* = 4]). Eighty-two of the deaths (79.6%) were determined at autopsy to have died from natural causes. The cause of death in the remaining 8 individuals (7.8%) was undetermined. There were no instances of suicide or carbon monoxide intoxication. Because the selection of our cases only included deaths in sauna rooms (where there is no bathtub in Korean jjimjilbangs), there were no instances of drowning. Of the 82 deaths caused by natural causes, 40 (38.8%) were attributed to ischemic heart disease, including acute myocardial infarction (*n* = 2). Thirty-eight cases were classified as other cardiac diseases, including suspicious conduction disorders and dilated cardiomyopathy. Six individuals had chronic alcohol-related disease based on pathological findings of chronic liver diseases, including liver cirrhosis. Acute alcohol intoxication was diagnosed as the cause of death in 4 cases based on BACs of >0.30% (0.32%, 0.33%, 0.34 and 0.38%, respectively). When the body position of the sauna of the subject was reported, 50 (48.6%) were in the supine position, 37 (35.9%) in the prone position, 10 (9.7%) in the side position, and 6 (5.8%) in the sitting position. The BAC was analyzed in all cases. Analysis of postmortem BAC showed that 81 (78.6%) individuals had alcohol in their blood. The mean BAC was 0.17% (range, 0.01–0.38%). Seventy-six individuals (73.8%) had a BAC of ≥0.08%. In 4 of the 15 female individuals, the mean BAC was 0.12%, and in 77 of the 88 male individuals, the mean BAC was 0.17%. The difference in mean BAC between men and women was statistically significant (*P* < 0.001). Decedents were classified into 2 groups according to their BAC: the NI group (BAC, <0.08%) and the I group (BAC, ≥0.08%).

Table 2 shows the comparisons of demographic and forensic characteristics of sauna deaths between the I and NI groups. Statistically significant differences were observed between the 2 groups for age, sex, and body position at the scene of death. There were more males in the I group than in the NI group (96.1% vs. 3.9%, respectively). The prevalence in the NI group peaked at 50–59 years, whilst that in the I group peaked at ≥60 years. No significant differences in the extent of obesity, coronary artery narrowing, and liver pathology were observed between the 2 groups. The I group contained a significantly greater proportion of individuals in the prone position at the scene of death (46.1%) than the NI group.

**Table 2:** Comparisons between the non-intoxication (NI) group and the intoxication (I) group

Characteristic	NI group	I group	Between group comparisons^a^	Trend analysis^b^
	(*n* = 27)	(*n* = 76)	*P*-value	*P*-value
Sex, *n* (%)				
Female	12 (44.4)	3 (3.9)		
Male	15 (55.6)	73 (96.1)	<0.001^**^	<0.001^**^
Age (years), *n* (%)				
< 29	0 (0.0)	2 (2.6)		
30–39	2 (7.4)	4 (5.3)		
40–49	5 (18.5)	24 (31.6)		
50–59	15 (55.6)	18 (23.7)		
≥ 60	5 (18.5)	28 (36.8)	0.033^*^	0.030^*^
Obesity, *n* (%)				
Underweight	1 (3.7)	3 (3.9)		
Normal	13 (48.2)	27 (35.5)		
Overweight	6 (22.2)	24 (31.6)		
Obese	7 (25.9)	22 (29.0)	0.713	0.449
Coronary artery narrowing, *n* (%)				
Minimal	14 (51.9)	36 (47.4)		
Mild	5 (18.5)	18 (23.7)		
Moderate	2 (7.4)	9 (11.8)		
Severe	6 (22.2)	13 (17.1)	0.839	0.960
Liver pathology, *n* (%)				
None	24 (88.9)	63 (82.9)		
Fatty liver disease	3 (11.1)	12 (15.8)		
Liver cirrhosis	0 (0)	1 (1.3)	0.818	0.395
Position, *n* (%)				
Supine	21 (77.8)	29 (38.2)		
Prone	2 (7.4)	35 (46.1)		
Side	2 (7.4)	8 (10.5)		
Sitting	2 (7.4)	4 (5.3)	<0.001^**^	0.047^*^

^*^P < 0.05,^**^P < 0.001

^a^Chi-square test or Fisher’s exact test

^b^Linear-by-linear association

Multinomial logistic regression analysis was performed to examine differences in characteristics between the I and NI groups (Table 3). Univariate multinomial logistic regression analysis showed that age, obesity, and coronary artery narrowing were not statistically significantly associated with BAC. Therefore, these variables were excluded from the multivariate multinomial logistic regression analysis. Using the NI group as a reference,multivariate multinomial logistic regression analysis showed that male sex (odds ratio [OR]: 17.4,*P* < 0.001) and the prone position at the scene of death (OR: 11.3, *P* < 0.05) were more prevalent in the I group than in the NI group. In contrast, no significant differences in the side or sitting positions at the scene of death were observed between the I and NI groups.

**Table 3:** Multivariate analysis of the influence of demographic and forensic characteristics on blood alcohol concentration (BAC)

Characteristic	BAC (reference category, NIgroup [*n* = 27])	
	I group (*n* = 76)	
	Crude OR (95.0% CI)^a^	Adjusted OR (95.0% CI)^b^
Sex		
Female	1	1
Male	19.47 (4.89–77.52)^**^	17.37 (3.78–79.76)^**^
Age (years)		
< 39	1	–
40–49	1.60 (0.25–10.36)	–
50–59	1.87 (0.29–12.01)	–
≥ 60	0.40 (0.07–2.28)	–
Obesity		
Underweight	1	–
Normal	0.69 (0.07–7.32)	–
Overweight	1.33 (0.12–15.20)	–
Obese	1.05 (0.09–11.75)	–
Coronary artery narrowing		
Minimal	1	–
Mild	1.40 (0.44–4.50)	–
Moderate	1.75 (0.34–9.13)	–
Severe	0.84 (0.27–2.66)	–
Liver pathology		
Absent	1	–
Present	1.65 (0.43–6.31)	–
Position		
Supine	1	
Prone	12.67 (2.74–58.61)^*^	11.30 (2.13–60.07)^*^
Side	2.90 (0.56–15.05)	2.52 (0.40–16.04)
Sitting	1.45 (0.24–8.66)	1.36 (0.18–10.46)

*CI* confidence interval, *I* intoxication, *NI* non-intoxication, *OR* odds ratio

^*^P < 0.05, ^**^P < 0.001

^a^Univariate multinomial logistic regression analysis

^b^Multivariate multinomial logistic regression analysis adjusted for sex and position

## Discussion

Fatalities arising from voluntary exposure to hot conditions indoors have been documented inside sweat lodges in the Australian outback and Finnish-style saunas [3–6, 9]. Much of the data on sauna deaths based on postmortem examinations have been reported in Finland and Sweden [3–6], due to their cultural tradition of sauna bathing. However, sauna deaths have also occurred in Korea. The most obvious explanation lies in the fact that jjimjilbangs have been a part of the modern Korean lifestyle since the 1980’s. Furthermore, most jjimjilbangs are open 24 h. They provide public sleeping rooms, as well as facilities for sauna bathing, at lower prices ($6–12) than the usual accommodation in Korea. In the daytime, most visitors are families. However, many Koreans visit jjimjilbangs after drinking or working overnight to sober up and to cure hangovers through sauna bathing or sleeping at night and in the early morning. Although there are isolated sleeping rooms and employees to guide patrons, some visitors lie down on the sauna floor to relax and sometimes fall asleep in the hot sauna rooms. Therefore, forensic pathologists in Korea have reported sauna deaths despite an extremely low medico-legal autopsy rate (1.6%) [10].

Binge drinking (a widespread pattern of alcohol intake accounting for >75.0% of alcohol consumption [10]) is defined by the National Institute on Alcohol Abuse and Alcoholism [11] as consuming >4/5 alcoholic beverages within 2 h, elevating the BAC to ≥0.08%. For Korean adults, drinking alcohol is a large part of social culture. According to a nationwide database [12], 81.6% of adult men and 52.4% of adult women are alcohol drinkers. Hence, alcohol consumption is a current major health concern and a source of socioeconomic burden in Korea [13]. In this study, positive BACs were found in 78.6% and the rate of intoxication (BAC ≥ 0.08%) was 73.8% overall (82.9% in men and 20.0% in women). Although previous studies on sauna deaths [4–6] reported that alcohol was detected in 50.0 to 84.0% of deceased individuals, there have been no studies on acute alcohol intoxication in sauna deaths. It is noteworthy that most of the alcohol detected in sauna deaths was at intoxication levels and that men were more significantly associated with intoxication. This difference in BAC may be explained by the fact that many Koreans, especially men, use saunas after drinking or working overnight to sober up and to cure hangovers. Thus, a high prevalence of alcohol consumption in men resulted in a sex difference in sauna deaths.

Alcohol intoxication may play a role in the cause of death in saunas. In an autopsy, the establishment of alcohol poisoning as a cause of death is mainly based on alcohol concentration in blood or urine. According to previous studies [14, 15], a BAC of 0.30% to more than 0.35% is commonly considered potentially fatal, and most deceased have a BAC of more than 0.40%. Subsequently, the accepted criteria for establishing a diagnosis of acute alcohol intoxication as a cause of death are dependent on a BAC of >0.30%. At this high dose, the alcohol itself exerts a sedative and depressive effect on neural cells in the respiratory center of the midbrain. Therefore, a putative mechanism of death by alcohol intoxication is considered suppression of respiratory function regulated by the medulla oblongata with concomitant central nervous hypoxia [16–18]. However, the cause-effect relationship between alcohol and death is ambiguous in sauna deaths and it is difficult to assess the significance of lower BACs of between 0.08 and0.29%. Therefore, although most studies on sauna deaths [4–6] have reported that alcohol consumption was detected in 50.0–84.0% of deceased individuals, the role of alcohol consumption in sauna deaths is underestimated, especially in cases with low BACs. In our series, 4 cases of acute alcohol intoxication were confirmed as the primary cause of death according to findings of a BAC of >0.30%. However, the criteria for establishing a diagnosis of acute alcohol intoxication as the cause of death should consider BAC and other possibilities in cases where individuals die within a few hours of binge drinking in specific environments, such as saunas. First, a plausible theory for individuals with a lower than normally accepted BAC for cause of death is that they may experience cerebral hypoxia caused by suppression of respiratory function in the sauna while alcohol metabolism proceeds until the moment of death. It is believed to be preceded by a loss of consciousness owing to binge drinking. Meanwhile, the alcohol is being metabolized prior to sauna death. The blood alcohol metabolism rate varies according to age, sex, body weight, BAC, and individual tolerance, yet heavy drinkers can metabolize >0.02 and 0.03% of alcohol per hour [14]. Second, body position at the scene of death may be associated with acute alcohol intoxication. In our series, the prone position (OR: 11.3, *P* < 0.05) was observed more frequently in the I group than in the NI group. Our data suggest that the prone position in those with a BAC of ≥0.08% may be meaningful, because intoxicated individuals would not be able to move out of that difficult position, compromising chest movement for respiration. Furthermore, hyperthermic environments, such as a hot sauna, induce hyperventilation and contribute to thermoregulatory heat loss responses and the internal alarm to evade the hot environment. The prone position in intoxicated individuals with a BAC of ≥0.08%could interfere with the hyperthermia-induced hyperventilation and escaping from the hot environment.

Although a high prevalence of alcohol consumption has continually been reported in sauna deaths [3–6, 19], the cause of death has been attributed to ischemic heart disease or hyperthermia (or is undetermined) in individuals with a relatively low BAC of <0.30%. Certainly the presence of coronary atherosclerosis and/or old myocardial infarction combined with exposure to heat may result in sauna deaths. However, the significance remains unclear. Moreover, a study by Hannuksela and Ellahham [20] suggested that for most people with coronary heart disease or a history of myocardial infarction, sauna bathing is safe, and sudden death of cardiac origin during sauna bathing is an extremely rare event. A recent study [1] also indicated that sauna bathing is associated with a significant reduction in the risk of fatal cardiovascular disease. Our data suggest that individuals with intoxication-level BACs who died in the sauna tended to be in the prone position at the scene of death, which may have affected respiratory function and led to death. With a comprehensive autopsy that incorporates a thorough investigation of the scene of death and risk factors of deceased individuals, including age and drinking history, forensic pathologists have suggested that acute alcohol intoxication is a potential cause of death, especially in individuals with a BAC of <0.30%.

This study has 2 limitations. First, in Korea, the decision to perform a medico-legal autopsy is made by public prosecutors employed by the Ministry of Government Administration and Home Affairs. Autopsies are typically only performed in cases of need, as in suspicious deaths associated with a crime. Therefore, the medico-legal autopsy rate was extremely low (1.6%) [10], preventing us from determining the mortality rate for sauna deaths per 100,000 of the Korean population. Furthermore, l sauna users that have suffered a medical emergency but are not deceased are transferred to a hospital. Based on the Korean medico-legal system, if they die subsequently an autopsy is not usually performed. Death certificates are issued by clinicians. Therefore, there are no records of in-hospital deaths associated with sauna bathing. Second, this study did not present exact diagnoses of past medical disorders based on the International Statistical Classification of Diseases and Related Health Problems (10th revision). Most of the information was derived from postmortem findings.

Despite these limitations, this study has 2 major strengths. To the best of our knowledge, this is the first study to classify sauna deaths into 2 groups according to BAC (NI and I groups) and to compare the characteristics of sauna deaths. The majority of previous studies [3–6, 19] have focused on the cause and manner of deaths in saunas; they reported that the cause of death in saunas were most commonly attributed to ischemic heart disease with a few deaths attributed to hyperthermia. Second, this is the first nationwide study of sauna deaths in Korea that has used representative data. It is especially noteworthy that this is the first collection of data for sauna deaths outside Finland and Sweden. New information related to diverse social and cultural backgrounds adds to the current knowledge. The forensic autopsy data that presents demographic and postmortem findings, including BACs, is especially meaningful in quantitative research focusing on sauna deaths.

## Conclusions

Saunas are usually used for relaxation and refreshment. The benefits of sauna bathing have been shown in epidemiological studies [1, 2]. However, the hazardous role of alcohol consumption in saunas has been not fully emphasized, especially at lower BACs; thus, the significance of the relationship between alcohol consumption and sauna death has been unknown. Our study suggests that sauna deaths have different characteristics in the I and NI groups, according to BAC at autopsy. The results presented herein support evidence that sauna death is still undoubtedly a diagnosis of exclusion. The cause of death of individuals with no alcohol detected in their blood may be attributed to heart disease, such as ischemic heart disease. However, individuals (especially men) with alcohol intoxication were associated with the prone position at the scene of death. This suggests that respiratory dysfunction caused by acute alcohol intoxication and body position could lead to sauna death despite lower BACs. Furthermore, considering the high prevalence of alcohol intoxication in sauna deaths, alcohol consumption may be the most significant risk factor for sauna death. Monitoring sauna bathing after alcohol consumption is a key step in preventing sauna deaths.

## Key points

1. A high prevalence of alcohol intoxication (blood alcohol concentration, ≥0.08%) is associated with sauna deaths.
2. Male sex and a prone position at the scene of death were significantly higher in the intoxication group than in the non-intoxication group of sauna deaths.
3. Alcohol consumption may be the most significant risk factor for sauna death.
4. Monitoring sauna bathing after alcohol consumption is a key step in preventing sauna deaths.
